# The use of FGF21 analogues in MASH and MASH cirrhosis – metabolic master regulators or liver-specific mechanisms?^☆^

**DOI:** 10.1016/j.jhepr.2026.101957

**Published:** 2026-07-11

**Authors:** A.G. Holleboom, Jörn M. Schattenberg

**Affiliations:** 1 Department of Internal Medicine, Amsterdam UMC, University of Amsterdam, 1105 AZ Amsterdam, Netherlands; 2 Department of Internal Medicine II, Saarland University Medical Center, 66421 Homburg, Germany; 3 Saarland University, 66123 Saarbrücken, Germany; 4 Pharma Science Hub, Saarbrücken, Homburg, Germany

**Keywords:** Metabolic modulation, anti-fibrotic activity, liver disease, adipose tissue, type 2 diabetes, clinical trials

## Abstract

Fibroblast growth factor 21 (FGF21) is a pleiotropic endocrine hormone that coordinates metabolic homeostasis across the liver, adipose tissue, pancreas, and central nervous system. In metabolic dysfunction-associated steatotic liver disease (MASLD), circulating FGF21 levels are markedly elevated, yet downstream signalling remains blunted – a state of FGF21 resistance driven by reduced β-klotho co-receptor availability and altered receptor complex function. Pharmacological FGF21 analogues were engineered to overcome the unfavourable pharmacokinetics of native FGF21 and achieve sustained target engagement at the FGFR1c/β-klotho complex in key tissues. Liver-directed effects of FGF21 analogues encompass suppression of *de novo* lipogenesis and lipotoxic intermediates, attenuation of hepatocellular stress and innate immune activation, and inhibition of hepatic stellate cell activation. Systemically, FGF21 signalling remodels adipose tissue via the FGF21–adiponectin axis, reduces atherogenic dyslipidaemia, improves insulin sensitivity, and modulates central pathways of energy expenditure and macronutrient preference. This breadth of action positions FGF21 analogues as both metabolic master regulators and liver-directed therapeutics. In phase II clinical trials, efruxifermin, pegozafermin, and efimosfermin have demonstrated meaningful histological improvements in patients with fibrotic metabolic dysfunction-associated steatohepatitis (MASH). In addition, efruxifermin has been shown to promote histological regression of cirrhosis in a dedicated trial of patients with compensated cirrhosis. Phase III programmes are now underway to establish their effects on liver-related outcomes and mortality. FGF21 analogues appear particularly potent in inducing fibrosis regression despite only modest weight loss, supporting a complementary combination strategy. Current evidence supports FGF21 pathway activation as a meaningful strategy to modify MASH progression and potentially reverse cirrhotic liver disease.

## Graphical abstract

**Figure:**
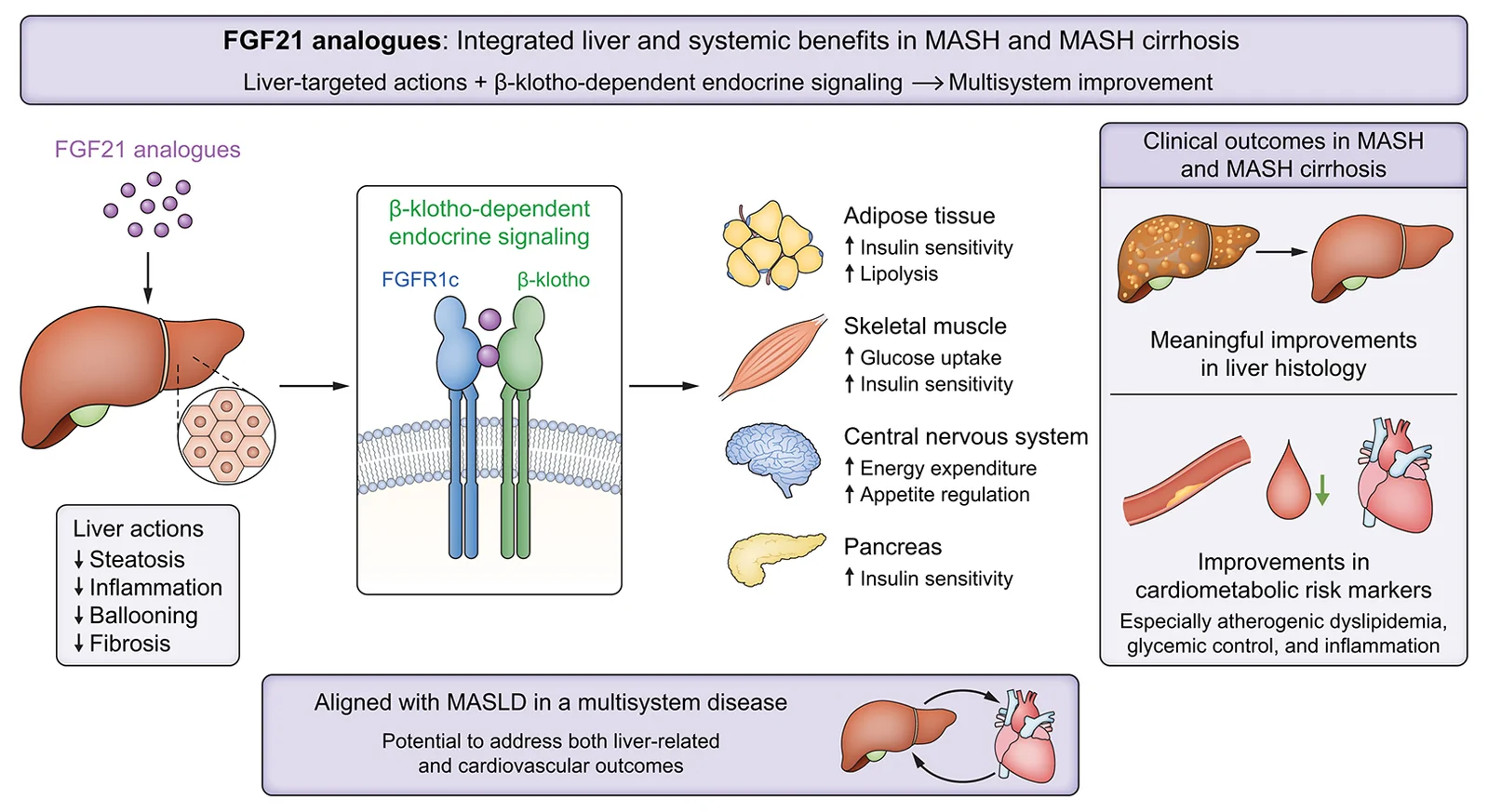

**Keypoints**

• FGF21 is a pleiotropic endocrine hormone acting as a stress-adaptive signal across liver, adipose tissue, pancreas, and the central nervous system.
• FGF21 exerts liver-directed effects by suppressing *de novo* lipogenesis, reducing lipotoxic intermediates and hepatocellular stress, attenuating innate immune activation, and inhibiting hepatic stellate cells.
• Systemic metabolic effects of FGF21 include adipose tissue remodelling via the FGF21–adiponectin axis, atherogenic dyslipidaemia reduction, insulin sensitisation, and central energy regulation.
• In phase II clinical trials, efruxifermin, pegozafermin, and efimosfermin demonstrated meaningful histological improvement in fibrotic MASH with favourable safety profiles.
• For efruxifermin, histological reversal of compensated MASH cirrhosis was shown in a landmark proof-of-concept study addressing the population with the greatest unmet need for pharmacotherapy.
• Metabolic and hepatic benefits occur independent of weight loss, distinguishing the class from incretin-based therapies.
• Complementary combination strategies seem feasible in the future.
• Phase III clinical trials are ongoing to establish the benefit on liver-related outcomes and the durability of the response.

## Introduction

Metabolic dysfunction–associated steatotic liver disease (MASLD) is now the most prevalent chronic liver disease worldwide, reflecting the global rise in obesity, insulin resistance, and type 2 diabetes mellitus (T2DM).[1], [2], [3] MASLD encompasses a spectrum from steatosis to metabolic dysfunction–associated steatohepatitis (MASH), which features hepatocellular injury, inflammation, and fibrogenesis.^4^^,^^5^ While steatosis alone may follow a relatively indolent course in many individuals, progression to MASH and advanced liver fibrosis confers markedly higher risks of cirrhosis, hepatocellular carcinoma, hepatic decompensation, and liver-related mortality.[6], [7], [8]

Fibrosis stage in the liver is the strongest predictor of long-term outcomes in MASLD/MASH, surpassing the prognostic value of steatosis grade or steatohepatitis.[8], [9], [10] Therefore, clinical pathways increasingly prioritise non-invasive identification of significant (F2) and advanced fibrosis (F3) among large numbers of individuals with metabolic risk,^11^ to identify those in need of therapeutic intervention and to prevent adverse outcomes.^12^ Second, the regulatory path towards accelerated approval of MASH therapies also relies heavily on assessments of effect on fibrosis, as well as on steatohepatitis as a driver of fibrogenesis. These measures of chronic liver injury plausibly decrease the risk of liver-related outcomes.[13], [14], [15]

The growing burden of MASH-related cirrhosis is reshaping the epidemiology of end-stage liver disease, with MASLD becoming a leading indication for liver transplantation in many regions.[16], [17], [18] Importantly, MASLD is a systemic disease tightly linked to cardiometabolic comorbidities, including obesity, T2DM, atherosclerotic cardiovascular disease, chronic kidney disease, and heart failure, which together account for most deaths in this population.[19], [20], [21], [22] This extrahepatic disease burden creates a therapeutic “double bind”: A drug that improves liver histology but has no impact on cardiometabolic comorbidities, such as cardiovascular disease and chronic kidney disease, is less likely to be cost-effective and meaningful in the long term, whereas an agent that improves cardiometabolic parameters in addition to reduction in MASH and fibrosis may prevent liver-related events and improve long-term outcomes.

Finding effective pharmacological therapies for fibrotic MASLD is still an area of intense development.^23^^,^^24^ Early drug development efforts often focused on targeting downstream inflammatory or fibrogenic pathways, reflecting paradigms established in other chronic liver diseases.^25^^,^^26^ Yet repeated late-stage failures and modest effect sizes for some single-pathway agents have reinforced the idea that MASH is not simply “hepatic inflammation plus fibrosis,” but rather the hepatic manifestation of multisystem metabolic dysfunction.^23^^,^^24^^,^[26], [27], [28] This has motivated a paradigm shift toward therapies that address upstream metabolic drivers – insulin resistance, adipose tissue dysfunction, dyslipidaemia, altered substrate flux, and energy imbalance – while still delivering measurable improvements in fibrosis.^23^^,^^24^^,^[26], [27], [28]

Within this evolving landscape, fibroblast growth factor 21 (FGF21) has emerged as a particularly attractive target. Initially identified as a fasting-induced hepatokine regulated by peroxisome proliferator-activated receptor (PPAR)α, FGF21 is now recognised as a pleiotropic endocrine signal coordinating metabolic homeostasis across the liver, adipose tissue, pancreas, and central nervous system.[29], [30], [31], [32], [33], [34], [35], [36] Pharmacological FGF21 analogues have demonstrated consistent reductions in hepatic steatosis, improvements in metabolic parameters – particularly atherogenic dyslipidaemia – and, critically, histological fibrosis regression, including in patients with compensated MASH cirrhosis.[37], [38], [39] Thus, FGF21 analogues are both “metabolic master regulators” and “liver disease modifiers”.^26^^,^^40^

This review explores the therapeutic effects of FGF21 analogues as systemic metabolic regulators and liver-directed therapeutics. We discuss the implications for clinical development, patient selection, combination strategies, and long-term outcomes – especially in the context of advanced fibrosis and compensated cirrhosis.

## FGF21 biology: Discovery, expression, and physiological role

### Discovery and classification of FGF21

FGF21 belongs to the endocrine subfamily of fibroblast growth factors, which also includes FGF19 and FGF23. Unlike paracrine FGFs, endocrine FGFs lack a heparin-binding domain, enabling them to circulate systemically and act as hormones.^33^^,^^34^^,^^41^ FGF21 was first identified in the early 2000s and subsequently linked to fasting adaptation through PPARα-dependent induction in liver, coordinating lipid handling, ketogenesis, and systemic metabolic responses.^29^^,^^30^^,^[32], [33], [34] Early gain-of-function studies also demonstrated that pharmacological FGF21 could lower glucose, improve insulin sensitivity, and reduce body weight in rodent models, establishing translational interest in FGF21 as a therapeutic candidate for metabolic disease.[32], [33], [34], [35]

From an evolutionary perspective, FGF21 appears to function as a stress-adaptive hormone that helps organisms cope with fluctuations in nutrient availability and macronutrient composition.^40^ This stress biology framework helps reconcile apparently paradoxical regulatory functions – FGF21 rises during fasting but can also increase in response to carbohydrate overfeeding and alcohol exposure – suggesting that FGF21 is activated when metabolic homeostasis is challenged rather than merely reflecting caloric deficit (Fig. 1).[29], [30], [31]^,^^33^^,^^34^^,^^42^

**Fig. 1:**
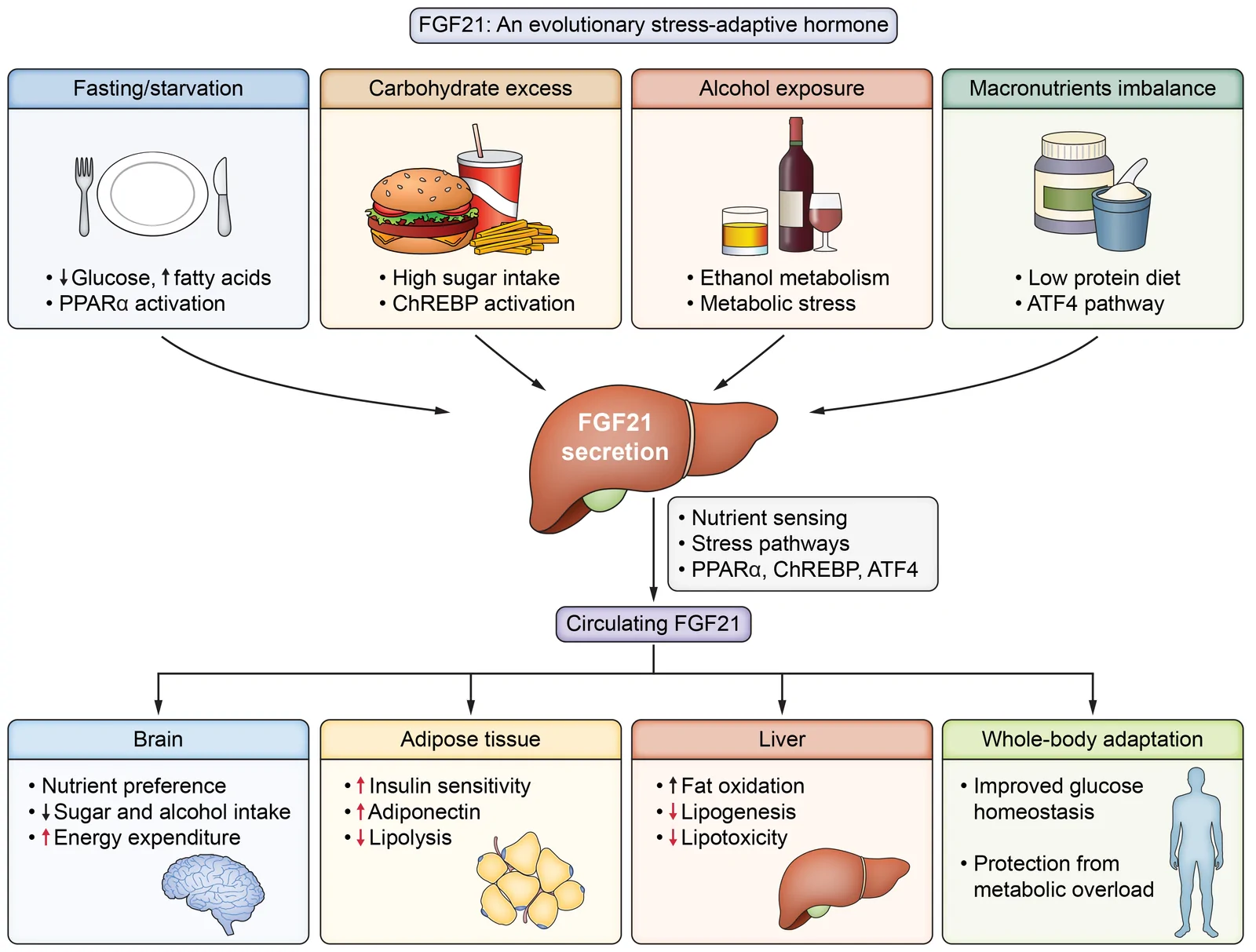
**FGF21**, **an evolutionary stress-adaptive hormone.**

### Tissue expression and regulation

The liver is the primary source of circulating FGF21 under most physiological conditions, accounting for the majority of plasma concentrations.[29], [30], [31]^,^^33^^,^^34^ Hepatocyte-derived FGF21 acts in an endocrine manner on distant tissues, including adipose tissue and the central nervous system.^33^^,^^34^ However, FGF21 expression is also observed in white and brown adipose tissue, pancreas, skeletal muscle, and, under certain conditions, the central nervous system, where it may exert autocrine or paracrine effects.^33^^,^^34^^,^^36^

FGF21 expression is highly responsive to nutritional and metabolic stress. Fasting induces hepatic *FGF21* transcription via PPARα activation, promoting fatty acid oxidation, ketogenesis, and gluconeogenesis.[29], [30], [31] In contrast, carbohydrate excess can induce FGF21 as part of a nutrient-stress programme, and short-term carbohydrate overfeeding in humans can markedly elevate circulating FGF21.^42^ Alcohol consumption also robustly induces hepatic FGF21, consistent with hepatocellular stress responses and altered redox balance; these observations align with broader evidence linking FGF21 to nutrient preference and alcohol intake regulation.^34^^,^^43^

This bidirectional inducibility suggests that FGF21 is best viewed as a signal of “metabolic strain” that triggers adaptive re-partitioning of substrates, modulation of energy expenditure, and behavioural adjustments in nutrient seeking and intake (Fig. 2).[33], [34], [35]

**Fig. 2:**
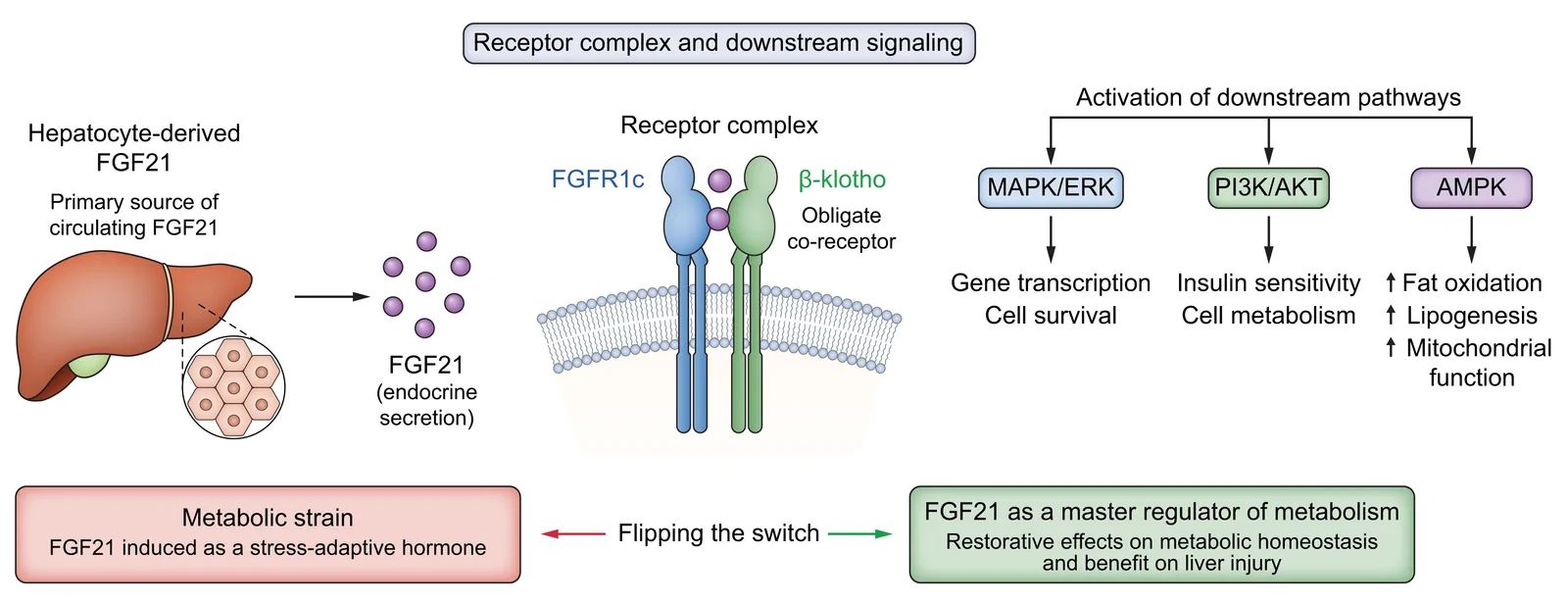
**FGF21**, **a master regulator of metabolic strain.**

### Circulating FGF21 levels and the concept of FGF21 resistance

Circulating FGF21 concentrations are elevated in obesity, T2DM, MASLD, and MASH, which may contribute to FGF21 resistance, a concept analogous to insulin or leptin resistance but not yet fully established in humans. This concept is supported by observations that, despite high circulating FGF21, downstream signalling and metabolic responses may be blunted in target tissues in obesity and related metabolic states.^44^^,^^45^ Mechanistically, reduced expression or availability of the obligate co-receptor β-klotho and/or altered receptor complex function in target tissues may limit downstream signalling despite high ligand concentrations.[44], [45], [46] Additional contributors may include chronic inflammation, altered FGF-R expression, and post-receptor signalling defects; there may be heterogeneity in “FGF21 resistance” across models and studies.^46^

For MASH therapeutics, “FGF21 resistance” has two major implications. First, high endogenous FGF21 levels do not necessarily indicate maximal pathway activation; rather, they may reflect a compensatory response to metabolic stress that is insufficient because receptor/co-receptor availability or downstream signalling is constrained.^46^ Second, pharmacological FGF21 analogues may overcome resistance by increasing effective exposure at the receptor complex, improving pharmacokinetics, and potentially stabilising the ligand-receptor interaction to amplify signalling despite reduced β-klotho expression.^46^ This framework helps explain why exogenous FGF21 analogues can remain effective even when baseline FGF21 is high.

### FGF21 signalling pathways and β-klotho–dependent tissue specificity

FGF21 exerts its biological effects by binding to fibroblast growth factor receptors (FGFRs), primarily FGFR1c (and in some contexts FGFR2c/3c), in the presence of the co-receptor β-klotho.[47], [48], [49], [50] β-Klotho is essential for high-affinity binding and signalling and confers tissue specificity by restricting FGF21 responsiveness to cells that co-express FGFRs and β-klotho.[47], [48], [49], [50]

β-klotho expression is highest in adipose tissue, liver, pancreas, and select regions of the brain.^49^ Upon receptor activation, FGF21 triggers intracellular signalling cascades including MAPK/ERK and other metabolic stress-response pathways, which regulate gene programmes involved in lipid metabolism, glucose uptake, mitochondrial function, oxidative stress defence, and inflammatory signaling.^31^^,^^33^^,^^34^^,^^36^^,^^41^ Tissue specificity matters: adipose tissue appears to be a major effector organ for several systemic metabolic actions of FGF21, and adipocyte β-klotho is required for many of its key *in vivo* signalling effects across multiple experimental models.^49^^,^^51^

This receptor biology provides a mechanistic basis for one of the central questions in MASH therapeutics: are the benefits of FGF21 analogues primarily systemic – mediated through adipose tissue remodelling, reduced lipid flux, and central regulation of energy balance – or liver-directed, reflecting reductions in intrahepatic stress and anti-fibrotic signalling? In reality, both are likely to contribute, and their relative contributions may differ by disease stage (*e.g*. earlier MASH *vs.* compensated cirrhosis) and patient phenotype (*e.g*. severe insulin resistance *vs.* prominent dyslipidaemia).^33^^,^^34^^,^^46^

## Liver-specific effects of FGF21 in MASLD and MASH

### Regulation of hepatic lipid metabolism

A consistent preclinical effect of FGF21 signalling is reduction of hepatic steatosis, driven by suppression of lipogenic pathways and induction of fatty acid oxidation/ketogenic pathways – features aligned with its fasting-adaptive role.[29], [30], [31]^,^^34^ FGF21 analogues therefore likely reduce both triglyceride accumulation and the burden of lipotoxic intermediates through enhanced mitochondrial capacities – thereby redu-cing steatohepatitis.^40^

### Anti-inflammatory effects and immune modulation

Indeed, in experimental models, FGF21 has been associated with reductions in hepatocyte stress signalling, oxidative stress, and inflammatory cytokine production, in parallel with reduced innate immune activation.^33^^,^^34^^,^^46^ By reducing the upstream triggers of inflammation (lipotoxicity and cellular stress), FGF21 signalling may “de-prime” Kupffer cells and recruited macrophages.^40^

### Antifibrotic actions and hepatic stellate cells

In turn, reduction in chemokine and cytokine signalling may diminish the paracrine activation of hepatic stellate cells, linking inflammation to fibrosis progression.^40^^,^^52^ The most clinically consequential proposed liver-directed effect of FGF21 signalling is its anti-fibrotic activity, including reduced hepatic stellate cell activation and decreased pro-fibrotic gene expression.^40^^,^^52^

Collectively, FGF21 signalling acts at multiple levels of MASH pathogenesis, enhancing lipid clearance, safeguarding hepatocytes from stress-induced injury, dampening apoptotic and inflammatory responses, and limiting fibrogenesis by inhibiting hepatic stellate cell differentiation. The relative importance of these mechanisms likely shifts across the disease spectrum: in early MASH, metabolic and inflammatory drivers may dominate; in compensated cirrhosis, reduction of ongoing injury and improvement in portal pressure determinants may be critical for clinically meaningful benefit.

## Systemic metabolic regulation by FGF21

### Central nervous system mechanisms and energy expenditure

FGF21 can act centrally to regulate energy balance and aspects of nutrient preference. Rodent and translational studies show that exogenous FGF21 improves insulin sensitivity and can increase energy expenditure through centrally mediated pathways.^53^^,^^54^ In the brain, FGFR1c and β-klotho are expressed in the hypothalamic suprachiasmatic nucleus, the dorsal vagal complex, and the amygdala, where they mediate the effects of FGF21 on the brain.^55^ In mechanistic work involving multiple murine models, central FGF21 signalling has been linked to sympathetic activation, glutaminergic neurons, leptin action and energy expenditure regulation, providing a plausible mechanism by which FGF21 analogues may influence weight, adipose tissue metabolism, and hepatic substrate flux.^55^^,^^56^ The underlying mechanisms include increased expression of thermogenic genes and enhanced sympathetic nerve activity in brown adipose tissue,^55^ leading to increased energy expenditure.^54^

### Adipose tissue effects and the adiponectin axis

Adipose tissue is an important peripheral target tissue for FGF21 signalling.^31^^,^^33^^,^^34^ Adipose tissue β-klotho expression is particularly important for insulin-sensitising and energy expenditure responses.^35^^,^^47^^,^^49^^,^^54^ A key downstream mediator of adipose tissue FGF21 signalling is adiponectin. Adiponectin has been identified as a downstream effector of FGF21 in regulating glucose homeostasis and insulin sensitivity, and the FGF21–adiponectin–ceramide axis has emerged as a major mediator of insulin action and energy expenditure.^36^^,^^41^ In a recent study, adipose tissue-specific overexpression of FGF21 protected mice against diet-induced obesity.^57^ These pathways provide a plausible mechanistic link between adipose tissue remodelling, reduced lipotoxic flux to the liver, and improvements in hepatic inflammation and fibrosis.^34^^,^^40^^,^^44^

### Effects on plasma lipids and cardiovascular risk

Across clinical trials, FGF21 analogues have consistently improved atherogenic lipid profiles, including triglycerides and apolipoprotein B-related lipid parameters (Table 2), alongside reductions in liver fat and improvements in glycaemic control. This lipid signature is particularly relevant for MASLD because cardiovascular disease is a dominant driver of mortality, and dyslipidaemia is a major modifiable risk factor in this population.[58], [59], [60] The clinical significance of these lipid improvements will ultimately depend on whether they translate to reduced cardiovascular events in long-term outcome trials. Nevertheless, given the strong epidemiologic link between MASLD and cardiovascular mortality, therapies that improve atherogenic dyslipidaemia while improving liver outcomes are likely to be favoured in real-world practice and guideline evolution.^61^

**Table 1:** Clinical trials of FGF21 analogues.

Compound	Company (acquisition by)	Phase III clinical trial name	Molecule	Results of Phase II clinical trial∗	ClinicalTrials.gov ID
Pegozafermin	89bio (Roche)	F2/F3: ENLIGHTEN fibrosis (n = 1,350) and Cirrhosis: ENLIGHTEN cirrhosis (n = 1,650)	GlycoPEGylation (single 20 kDa polyethylene glycol attached via glycan linkage) with mutations: N-terminal methionine + two point mutations to resist proteolysis and optimise FGFR1c/β-klotho binding) 30 mg qw and 44 mg q2w in non-cirrhotic MASH and compensated cirrhosis	ENLIVEN study; week 24 placebo corrected change for fibrosis improvement (50 mg) 21%.	NCT06318169 NCT06419374
Efruxifermin (EFX)	Akero Therapeutics (Novo Nordisk)	F2/F3 SYNCHRONY Histology (n = 1,650) Cirrhosis: SYNCHRONY Outcomes (n = 1,150) SYNCHRONY Real World (n = 700)	FGF21 domain fused at C-terminus to human IgG1 Fc via a flexible peptide linker; forms a homodimer (∼130 kDa) Point mutations for protease resistance and optimised FGFR1c/β-klotho activation; half-life ∼5-6 days EFX 28 mg and 50 mg qw s.c. MASH F2-3, cirrhosis	HARMONY-study; week 96 placebo corrected change for fibrosis improvement (50 mg) 30%	NCT06215716; NCT06528314; NCT06161571
Efimosfermin alfa (BOS-580)	Boston Pharma (GSK)	F2/F3: ZENITH-1 (n = 1,200) ZENITH-2 (n = 1,250) Cirrhosis: NEBULA-1 (n = 1,740) and NEBULA-2 (n = 380)	FGF21 domain fused to human IgG1 Fc C-terminal fragment via GS linker; forms a covalent dimer (∼89 kDa monomer) Mutations (*e.g*., Ala14/15, Cys27/120, intra-chain disulfides Cys252-Cys345); N-glycosylation at Asn77 for half-life extension (∼21 days, monthly s.c. dosing)	Phase 2b study week 24 placebo corrected change for fibsosis improvement (300 mg q4w) 24%	NCT07701993, NCT07704892, NCT07221227; NCT07221188

MASH, metabolic dysfunction-associated steatohepatitis; s.c., subcutaneous. ∗no head-to-head trials and crosstrial comparison of placebo corrected fibrosis improvement is not valid,

## Interaction with other hormonal systems and rationale for combination therapy

FGF21 intersects with broader endocrine networks (including insulin/glucagon axes and central appetite pathways), supporting interest in combination regimens with incretin-based therapies, particularly where weight loss and cardiometabolic risk reduction are major goals.^62^^,^^63^

From a practical standpoint, FGF21 analogues and incretin therapies may have complementary strengths. Incretins (*e.g*. glucagon-like peptide 1 [GLP-1] receptor agonists) drive substantial weight loss and improve glycaemic control; FGF21 analogues appear particularly potent in improving dyslipidaemia and may induce improvement of MASH fibrosis even with modest weight loss. This complementarity supports an emerging "metabolic combination" paradigm in which multiple upstream drivers are targeted simultaneously to maximise histological response at the level of the liver, while reducing systemic cardiometabolic risk.

### FGF21, feeding behaviour, and alcohol metabolism

FGF21 contributes to regulation of macronutrient preference and can suppress sweet and alcohol preference in preclinical models and non-human primates.^42^^,^^43^^,^^53^ Circuit-level studies further support a neurobiological pathway through which FGF21 reduces alcohol intake.^54^^,^^64^ These observations have translational relevance given the overlap between metabolic dysfunction and alcohol exposure,^65^ and they have motivated interest in FGF21-pathway therapeutics for both alcohol-related liver disease (ALD) and metabolic dysfunction- and alcohol-associated liver disease (MetALD). A therapy that simultaneously reduces alcohol preference and improves metabolic dysfunction could offer unique therapeutic value.

## Clinical development of FGF21 analogues

### Why analogues were needed: pharmacokinetics, exposure, and target engagement

The clinical development of FGF21-based therapies required substantial molecular engineering to overcome unfavourable pharmacokinetic properties of native FGF21. Endogenous FGF21 has a short circulating half-life and is susceptible to proteolytic degradation, rendering it unsuitable for chronic therapeutic use.^34^ Consequently, multiple long-acting FGF21 analogues have been developed using strategies such as PEGylation and Fc-fusion to enhance stability and duration of action while preserving β-klotho–dependent receptor activation.^34^^,^^40^

In therapeutic terms, the key requirement is sustained and sufficient “target engagement” at the FGFR1c/β-klotho complex in relevant tissues (notably adipose tissue and liver).^47^^,^^48^^,^^66^ Differences in molecular design may influence potency, receptor bias, tissue distribution, and tolerability. These differences partly explain why the FGF21 class contains multiple candidates with overlapping but not identical metabolic and hepatic profiles.^40^

### Early human proof-of-concept: Metabolic effects and class signals

Before large MASH histology trials, early clinical work with FGF21 analogues and related agonists established that pharmacological FGF21 pathway activation could improve metabolic parameters in humans, including insulin sensitivity and lipid profiles. These studies were important because they demonstrated translation of key metabolic actions from animal models to humans – particularly triglyceride lowering and improvements in dyslipidaemia – supporting the plausibility of downstream liver benefits in MASLD/MASH populations.[66], [67], [68]

### FGF21 analogues in MASH and MASH cirrhosis

Pegbelfermin, a PEGylated FGF21 analogue, was among the first therapeutics trialled for MASH, but it failed to meet its primary histological endpoints in the phase IIb FALCON-1^69^ and FALCON-2^70^ trials in patients with bridging fibrosis and compensated cirrhosis, respectively. The histological regression of fibrosis and resolution of MASH did not achieve statistical significance *vs.* placebo despite favourable trends in liver fat reduction and other non-invasive biomarkers. These results, together with a relatively modest and non–dose-dependent treatment effect, led to discontinuation of its clinical development in MASH.

At present, three long-acting FGF21 analogues are under clinical development at different trial stages, which we will now discuss, in full awareness that cross-trial comparisons are inherently limited. Efruxifermin (EFX), an Fc-FGF21 fusion protein with once weekly dosing, was shown to significantly improve hepatic histology in MASH with stage F2–F3 fibrosis, as well as compensated cirrhosis. In a phase IIb program, EFX increased the proportion of patients achieving fibrosis improvement without worsening of MASH and improved metabolic parameters such as lipids and glycaemic markers (Table 1).^71^ Importantly, after 96 weeks of treatment in patients with compensated cirrhosis, EFX was the first compound to demonstrate the histological regression of cirrhosis in parallel to the improvement of biomarkers in the Symmetry trial.^39^ EFX is currently being evaluated in three clinical trials within the SYNCHRONY programme (Table 2). The SYNCHRONY Histology trial (NCT06215716) is investigating its efficacy in patients with non-cirrhotic MASH, with the primary aim of demonstrating superiority in achieving the histological endpoint at 52 weeks to support conditional approval by the US Food and Drug Administration (FDA). The SYNCHRONY Outcomes trial (NCT06528314) is designed to determine whether EFX reduces the incidence of clinically significant portal hypertension and major adverse liver outcomes. Finally, the SYNCHRONY Real World study (NCT06161571) is evaluating the safety and tolerability of EFX in patients with phenotypic MASLD identified using non-invasive biomarkers, providing insights into its potential use in routine clinical practice (see Table 3).

**Table 2:** Phase II results.

Endpoint	HARMONY (F2–F3, week 24) EFX	SYMMETRY (F4, week 96) EFX	ENLIVEN trial (F2–F3 MASH, week 24) Pegozafermin	Efimosfermin alfa (BOS-580) (F2–F3 MASH, 300 mg monthly s.c., 24 weeks, n = 84)
MASH resolution (no fibrosis worsening)	58% (50 mg) *vs.* 19% placebo	24% *vs.* 10% placebo	2% (placebo), 23% (30 mg qw) and 26% (44 mg q2w)	68% *vs*. 29% (placebo)
Fibrosis regression (≥1 stage, no MASH worsening)	42% (50 mg) *vs.* 12% placebo	39% *vs.* 15% placebo	7% (placebo), 26% (30 mg qw) and 27% (44 mg q2w)	45 *vs.* 21%
Weight loss (%)	8-10%	7-9%	6-7%	6-8%
HbA1c reduction	-0.7%	-0.6%	-0.5 and -0.6%	-0.8% to -1.0%
Triglyceride decrease	35% - 45%	40%	45-50%	40-50%
LDL change	-8%	-15%	+2-4%	-5 to -10%

**Table 3:** Safety and AEs.

Adverse event	Efruxifermin (HARMONY/SYMMETRY)	Pegozafermin (ENLIVEN)	Efimosfermin (Phase II F2–F3)
Any TEAE	90-95%	85-90%	75-80%
GI disorders (diarrhoea/nausea)	Diarrhoea 50%, nausea 45%	Diarrhoea 20-30%, nausea 15-25%	Diarrhoea 30-40%, nausea 25%
Injection site reactions	25-30% (bruising/erythema, mild)	10-15%	15-20%
Discontinuation	3-5% (GI-related)	2-4%	5% (GI/pruritus)
Serious AEs	0-2% (none drug-related)	1%	0%
Deaths	0% (no deaths in HARMONY n = 128; SYMMETRY n = 181)	0% (no deaths reported)	0% (no deaths reported)
ADA incidence	55% (low titre, no neutralizing Ab impact reported)	<10%	Not reported
Liver safety	No DILI/decompensation	No DILI	No DILI

Note that trials of efruxifermin enrolled patients with MASH in the stages F2–F3 and MASH cF4, while the other programmes only enrolled patients with non-cirrhotic MASH.

ADA, anti-drug antibody; AE, adverse event; DILI, drug-induced liver injury; MASH, metabolic dysfunction-associated steatohepatitis; TEAE, treatment-emergent adverse event.

Pegozafermin (PGZ) is a glycoPEGylated analogue of human FGF21 that allows for weekly subcutaneous dosing. In the phase IIb ENLIVEN study, weekly and biweekly dosing achieved comparable, positive effects on the primary histological endpoints in patients with fibrotic MASH at week 24 (Table 1). In a small *post hoc* analysis of 12 patients with compensated cirrhosis enrolled in ENLIVEN, 5 of the 11 patients who received PGZ showed regression to a pre-cirrhotic stage by week 24. In addition to its evaluation in MASH, PGZ has also been investigated for severe hypertriglyceridaemia.^72^ The phase III clinical programme is evaluating longer treatment durations in both non-cirrhotic and cirrhotic MASH, with the first results expected in 2027 and 2028.

In contrast to other FGF21 analogues requiring weekly dosing, efimosfermin (EFI) has been engineered as a covalent dimer with stabilising mutations that extend its half-life to approximately 21 days, allowing once-every-4-week dosing. In the phase II program, the effects on MASLD assessed with non-invasive parameters (MRI-proton density fat fraction) were at a level comparable to other FGF21 analogues.^73^ The histological readout after week 24 suggested a benefit with a placebo-corrected delta of 24% for achieving improvement of fibrosis by one stage in non-cirrhotic MASH (Table 1).^74^ Based on these data, a phase III program in fibrotic and cirrhotic MASH is expected.

Overall, the effects on metabolic parameters, including lipids, glycated haemoglobin, and body weight, were not particularly pronounced across the different trials and treatment durations, supporting their future use primarily as liver-directed therapies.

## Comparative positioning, limitations, and future directions

### Positioning within the evolving MASLD/MASH therapeutic landscape

FGF21 analogues have emerged in a competitive therapeutic landscape for fibrotic MASH, which includes incretin-based therapies, dual and triple hormone agonists, a pan-PPAR agonist, thyroid hormone receptor-β agonists, and inhibitors of *de novo* lipogenesis.^26^^,^^61^ FGF21 analogues are distinguished by the breadth of biological effects observed across trials: large reductions in liver fat, improvements in lipid profiles, and histologic signals consistent with fibro-sis improvement.^40^

Analogous to the thyroid hormone receptor beta agonist resmetirom,^75^ FGF21 analogues may deliver meaningful hepatic responses even when weight loss is absent or modest. For FGF21 analogues, this supports the view that adipose remodelling, lipid repartitioning, and direct hepatic stress reduction contribute to efficacy.^33^^,^^34^^,^^46^ In contrast, incretin therapies (*e.g*. semaglutide) have shown strong weight loss and improvements in metabolic risk, and they are likely to become backbone therapies for many patients with MASLD because they address its main drivers, *i.e*. obesity and T2DM directly.^28^^,^^63^ The question may ultimately become not whether to use FGF21- or incretin-based therapies, but how best to combine these metabolic mechanisms to maximise both hepatic and systemic benefits.^24^^,^^62^ Looking ahead in the drug development pipeline, peptides with dual FGF21 and GLP-1 receptor activity exemplify the potential of combining these therapeutic approaches, and the first clinical trials are already underway.

### Limitations: surrogate endpoints, heterogeneity, and implementation

Key limitations remain. First, the durability of histologic response and effects on hard clinical endpoints (decompensation, transplantation, mortality) require confirmation in long-term outcome trials.^15^ Although fibrosis regression and MASH resolution are accepted surrogate endpoints in development pathways, the degree to which they can be captured with non-invasive biomarkers and the degree to which short-term histologic change predicts long-term hard liver-related outcomes may vary.^15^ Data on liver-related outcomes are expected from the ongoing phase III trial in patients with cirrhosis. Also, interindividual variability in response likely reflects heterogeneity in dominant pathophysiological drivers (visceral adiposity, glycaemic control, alcohol co-exposure) and tissue-level receptor/co-receptor biology (β-klotho expression), which may influence pharmacodynamic “target engagement” and magnitude of response. This variability suggests the need for precision tools to identify responders and to select rational combinations for non-responders.^35^^,^^62^

### Safety and tolerability

Across clinical trials (Table 1), FGF21 analogues have demonstrated a generally favourable safety and tolerability profile, with discontinuation rates of approximately 5% at 24 weeks and up to 17% at 96 weeks.[37], [38], [39] Common treatment-emergent adverse events include gastrointestinal symptoms (*e.g.* nausea and diarrhoea), which are generally transient and occur predominantly during treatment initiation. Injection-site reactions are also common, with most events being mild to moderate and occurring early during therapy. Importantly, rates of serious adverse events have generally been comparable between FGF21 analogue-treated groups and placebo in randomised trials, including studies involving patients with advanced fibrosis and compensated cirrhosis.[37], [38], [39]

In compensated cirrhosis populations, careful attention is typically paid to signals of hepatic decompensation, renal dysfunction, worsening ascites, or bleeding risk – events that would raise concern about haemodynamic instability or impaired hepatic reserve. To date, available data have not identified consistent safety signals in patients with compensated cirrhosis, though longer follow-up is required.^39^

Long-term safety beyond phase II/IIb horizons remains limited, and continued surveillance is warranted for potential class-related effects on bone metabolism and other endocrine axes observed as “signals” in earlier FGF21-class development programmes.^68^ Preclinical studies with native FGF21 demonstrated increased bone resorption, reduced osteoblast activity, and net bone loss through PPARγ-mediated pathways, raising theoretical concerns regarding chronic pharmacological FGF21 exposure.^68^ Whether these effects persist with newer analogues, and whether they remain clinically meaningful during long-term treatment, will only be clarified through long-term follow-up in phase III trials and post-marketing pharmacovigilance. Although native FGF21 promotes net bone loss, no changes in bone mineral density have been observed in published clinical trial data to date. Whether this favourable skeletal safety profile is maintained with long-term FGF21 analogue therapy – particularly in older patients with obesity, diabetes, cirrhosis, or pre-existing osteopenia – will require clarification through extended follow-up in phase III trials.

From a clinical monitoring standpoint, if FGF21 analogues enter routine care, pragmatic safety monitoring would likely include: (i) gastrointestinal tolerability early after initiation, (ii) routine liver chemistry and synthetic function panels (especially in advanced fibrosis/cirrhosis), (iii) lipid profiles to document pharmacodynamic response, and (iv) consideration of bone health assessment in long-term users pending definitive safety data. These monitoring elements align with broader best practices for metabolic therapies in populations with systemic comorbidity and variable hepatic reserve.

### Future directions: Biomarkers, combination regimens, and broader indications

1. **Biomarkers of response and resistance.** Candidate biomarkers include baseline metabolic phenotype (lipids, insulin resistance severity), circulating FGF21 levels, dynamic changes in lipidomics and proteomics, and markers linked to β-klotho biology. A major goal is to develop a “target engagement to outcome” chain: early pharmacodynamic signals (*e.g.* triglyceride lowering, adiponectin changes) that predict later histologic response and, ultimately, clinical outcomes.
2. **Combination trials designed to assess synergy.** Trials explicitly testing FGF21 analogue plus GLP-1–based therapy are conceptually appealing because they combine strong weight loss and glycaemic improvement with potent lipid and liver fat effects, potentially maximising fibrosis regression and cardiometabolic risk reduction.
3. **Outcomes in compensated cirrhosis.** For cirrhotic MASLD, ongoing studies are currently exploring the development of clinically significant portal hypertension and related complications. As routine measurement of the hepatic venous pressure gradient is not feasible in large phase III trials, validated non-invasive surrogates of clinically significant portal hypertension are used to identify patients requiring confirmatory diagnostic procedures. In addition, liver-related clinical events are being captured as key endpoints to support full regulatory approval.
4. **MetALD/ALD overlap populations.** Given the role of FGF21 in alcohol use and the increasing recognition of mixed aetiologies, expansion into MetALD/ALD overlap indications is biologically plausible and clinically important.

## Conclusion

FGF21 analogues represent a promising therapeutic advance in the treatment of MASH and MASH cirrhosis. Their efficacy reflects an integration of liver-specific actions and systemic metabolic regulation enabled by β-klotho–dependent endocrine signalling. Across multiple clinical programmes, FGF21 analogues have produced meaningful improvements in liver histology and in cardiometabolic risk markers – particularly atherogenic dyslipidaemia. These effects align well with MASLD as a multisystem disease in which liver-related and cardiovascular outcomes co-occur.

The extension of FGF21 analogues into compensated cirrhosis – tested in dedicated trials – marks a pivotal advance in attempts to modify advanced disease biology rather than only prevent progression. This is particularly important because compensated cirrhosis represents an important intervention point: preventing transition to decompensation yields outsized clinical benefit, yet historically pharmacotherapy has struggled to halt or regress cirrhosis. While longer-term data are needed to demonstrate reductions in decompensation events and other hard outcomes, the available signals support the concept that targeting upstream metabolic stress can yield measurable improvements even in compensated cirrhosis.

As MASLD/MASH therapeutics move toward outcome-driven trials and combination strategies, FGF21 analogues are well positioned, particularly for patients with concomitant dyslipidaemia. Future work should clarify optimal patient selection, biomarkers of response and resistance (including β-klotho biology), durability of histologic benefit, and the most effective combination regimens – especially with incretin-based therapies that address obesity and T2DM robustly. Nevertheless, current evidence supports the concept that targeting metabolic master regulators such as FGF21 can meaningfully alter key biological drivers of MASH progression and may ultimately reshape how clinicians treat advanced metabolic liver disease.

## Abbreviations

ALD, alcohol-related liver disease; EFI, efimosfermin; EFX, efruxifermin; FGF, fibroblast growth factor; FGF, fibroblast growth factor; FGFR, fibroblast growth factor receptor; FGFR1c, fibroblast growth factor receptor 1c; GLP-1, glucagon-like peptide-1; MASH, metabolic dysfunction-associated steatohepatitis; MASLD, metabolic dysfunction-associated steatotic liver disease; MetALD, metabolic dysfunction- and alcohol-associated liver disease; PPAR, peroxisome proliferator-activated receptor; PGZ, pegozafermin; T2DM, type 2 diabetes mellitus.

## Authors’ contributions

Both authors drafted and wrote the article.

## Declaration of generative AI and AI-assisted technologies in the writing process

During the preparation of this work the authors used Claude (Anthropic) to improve language and readability of selected sections. After using this tool, the authors reviewed and edited the content as needed and take full responsibility for the content of the published article.

## Financial support

10.13039/501100007751AGH has received funding within the 10.13039/100020098Innovative Health Initiative Joint Undertaking (10.13039/501100004907IHI 10.13039/501100014757JU) GRIP on MASH (Global Research Initiative for Patient Screening on MASH) EU grant number 101132946. JMS has received funding within the 10.13039/100020098Innovative Health Initiative Joint Undertaking (10.13039/501100004907IHI 10.13039/501100014757JU) LIVERAIM under grant agreement No 101132901 and GRIP on MASH (Global Research Initiative for Patient Screening on MASH) EU grant number 101132946.

## Conflicts of interest

AGH declares consultancy for, Novo Nordisk, Echosens, Inventiva and Logick Energetics. JMS declares consultant honorary from Akero, Alexion, Altimmune, Astra Zeneca, 89Bio, Boehringer Ingelheim, Gilead Sciences, GSK, Ipsen, Inventiva Pharma, Kríya Therapeutics Madrigal Pharmaceuticals, Merck, Echosens, Eli Lilly, E-Therapeutics, Merck, MSD Sharp & Dohme GmbH, Novo Nordisk, Roche, Vantage Biosciences LtD; Speaker honorarium from AbbVie, Boehringer Ingelheim, Gilead Sciences, Ipsen, Lilly, Madrigal Pharmaceuticals, MSD, Novo Nordisk, Travel Support: Boehringer Ingelheim, IPSEN. Stockholder options: Hepta Bio, HeapCure Pharma.

Please refer to the accompanying ICMJE disclosure forms for further details.
